# Evaluating the protective effects of Aurodox in a murine model of Shiga toxin-producing *Escherichia coli*

**DOI:** 10.1038/s44259-025-00094-3

**Published:** 2025-04-01

**Authors:** Rebecca E. McHugh, Liam M. Rooney, David R. Mark, Kabo R. Wale, Megan Clapperton, Gail McConnell, Paul A. Hoskisson, Gillian R. Douce, Andrew J. Roe

**Affiliations:** 1 School of Infection and Immunity, University of Glasgow, Glasgow, UK; 2 Strathclyde Institute of Pharmacy and Biomedical Sciences, University of Strathclyde, 161 Cathedral Street, Glasgow, UK; 3 Biological Sciences, University of Botswana, Gaborone, Botswana; 4 Present Address: School of Infection and Immunity, University of Glasgow, Glasgow, UK

**Keywords:** Drug discovery, Microbiology

## Abstract

Shiga Toxin-Producing *E. coli* (STEC) are a group of acute small intestine pathogens responsible for foodborne outbreaks of bloody diarrhoea. The expression of Shiga toxins (Stx) carried by STEC can initiate Haemolytic Uremic Syndrome (HUS), a major cause of acute renal failure in children. Here, we investigate the anti-virulence potential of Aurodox - a natural product of *Streptomyces goldiniensis*. Previously, we have shown that Aurodox downregulates the expression of the T3SS, inhibiting epithelial cell colonisation in vitro. Here, we use the *Citrobacter rodentium* DBS770 (Cr Stx2_dact_) model of STEC infection to demonstrate that Aurodox protects mice against *Citrobacter rodentium-*associated colonic hyperplasia and Stx-mediated renal injury. Given antibiotic-associated dysbiosis of the gut is associated with inflammation and the emergence of opportunistic pathogens, we examined the effect of Aurodox on the faecal bacteriome. We show that although the microbial community is altered following Aurodox treatment, changes are distinct from those associated with traditional antibiotic therapies.

## Introduction

Shiga-Toxin Producing *Escherichia coli* (STEC) are a group of bacterial pathogens responsible for foodborne outbreaks of diarrhoeal disease^1^. These pathogens carry phage-encoded Shiga toxins (Stx), which are potent virulence factors capable of damaging the systemic vasculature^2^, resulting in acute renal damage in 5–15% of patients. This condition is known as haemolytic uremic syndrome (HUS)^1,3^ which has a mortality rate of 3–5%. As the natural reservoir of most STEC serotypes is the ruminant gut, disease outbreaks were classically associated with beef products or contaminated milk^4^. However, in recent years, the globalised nature of food supply chains has led to changes in STEC epidemiology, with disease often occurring in large outbreaks typically associated with produce cultivated with contaminated water^5,6^. Examples include the European O104:H4 outbreak of 2011 which resulted in 3950 cases and 53 deaths^7,8^.

Shiga toxin expression is tightly controlled by the bacterial SOS response, which is activated in response to DNA damage^9^. Therefore, the use of traditional antibiotics – which damage DNA and activate this type of response are not recommended for the treatment of STEC infections^10^. However, with improvements to the rate and accuracy of STEC diagnosis, there is now an opportunity to medically intervene in the critical pre-HUS window with non-antibiotic solutions. We propose that antivirulence (AV) drugs may provide an appropriate treatment option for STEC^11,12^. These drugs do not kill or inhibit the metabolic processes of the infecting bacteria but block a fundamental virulence factor to prevent disease in the host. Many enteric pathogens including STEC deploy one such factor, the Type III Secretion System (T3SS) for host colonisation^13^. In STEC, Enteropathogenic *E. coli* (EPEC) and the murine pathogen *Citrobacter rodentium*, the T3SS is encoded by the Locus of Enterocyte Effacement (LEE) and is essential for attachment and effacement of the epithelial cells of the gut^14,15^.

**Fig. 1:**
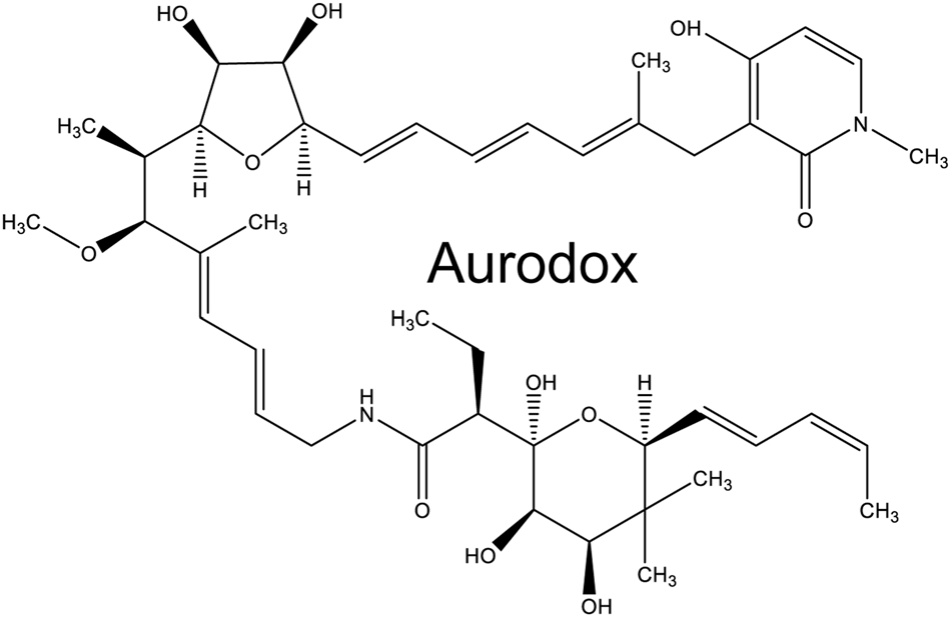
Structure of Aurodox. Chemical structure of hybrid polyketide/non-ribosomal peptide from Streptomyces goldiniensis, Aurodox.

The reliance of these pathogens on the T3SS to disease makes this structure an excellent target for antivirulence therapies. Many inhibitors of the T3SS have been identified including both synthetic small molecules and natural products^16–21^. In previous work, we have investigated Aurodox, a polyketide molecule derived from *Streptomyces goldiniensis* (Fig. 1)^22^. Aurodox was originally discovered in 1973, and investigated for its bactericidal effect against Gram-positive organisms but has never been licenced for use in the clinic^23^. In 2011, Aurodox was identified from a high throughput compound screen as a potential inhibitor of the T3SS^17^. Our recent work has shown that Aurodox strongly inhibits the T3SS in multiple Gram-negative pathogens including STEC, EPEC, *C. rodentium* and *Salmonella* Typhimurium (SPI-2)^21^. We have demonstrated that Aurodox exhibits its effects at the transcriptional level, by inhibiting T3SS expression, without inducing *recA*^22^. We have also investigated the biosynthesis of Aurodox in its native producer *S. goldiniensis*, creating a heterologous expression system capable of providing Aurodox for future studies^24,25^. This work has highlighted Aurodox as a highly potent T3SS inhibitor, with the potential for use in the clinic as a treatment for STEC.

To gauge the true potential of Aurodox as an AV therapy for STEC, it was essential to determine whether inhibition of the T3SS ultimately prevented the progression of intestinal disease to HUS. Therefore, a suitable animal model of infection was required. As most strains of human intestinal *E. coli* do not cause disease in mice, we challenged mice with an engineered strain of the natural murine pathogen *C. rodentium*, carrying a Stx-encoding prophage (Cr Stx2_dact,_ Cm^R^). As *C. rodentium* deploys the LEE-encoded T3SS for colon colonisation, and the endothelial cells of murine kidneys constitutively express the Stx receptor glycosphingolipid (Gb3), infection with Cr Stx2_dact_ provides a robust model of STEC-mediated HUS. This model, originally described by Mallick et al., recapitulates HUS phenotypes including acute kidney damage, severe weight loss and induction of Stx-mediated cytokines^26^. This model has been used effectively in several subsequent studies^27–29^.

Here, we used the Cr Stx2_dact_ model of STEC-HUS to test the antivirulence capacity of Aurodox. We demonstrated that Aurodox protects mice against both intestinal and systemic effects of STEC, including HUS-associated renal damage. In addition, we examined the effect of Aurodox on the gut microbiota in both infected and uninfected mice, demonstrating that a bloom in probiotic *Bifidobacterium animals* and *Akkermansia muciniphila* in response to Aurodox drives microbiome modulation but, in contrast to traditional antibiotics has minimal effects on diversity. These results nominate Aurodox as a candidate therapeutic for STEC infections in humans.

## Results

### Aurodox treatment reverses weight loss and improves survival of BALB/c mice challenged with *C. rodentium* DBS770 (Stx2_dact_)

In our previous studies, we characterised the inhibitory effects of Aurodox on Type III Secretion in STEC, EPEC and *C. rodentium* in vitro, demonstrating via GFP-reporter assays and protein secretion analysis that Aurodox abolishes T3SS at 5 µg/ml without affecting growth. We also showed that Aurodox blocks *E. coli* O157:H7 from colonising epithelial cells - thus diminishing attachment and effacement (A/E) lesion formation. It was therefore hypothesised that through inhibiting T3SS expression, Aurodox could prevent epithelial cell (A/E) in vivo, blocking colonisation and subsequent progression to HUS. This theory was previously explored by Kimura et al., who demonstrated that Aurodox blocked EPEC T3SS activity, and aurodox treatment improved survival in a lethal model of *C. rodentium infection* in C3H/HeJ mice^17^. However, the potential of Aurodox to protect mice against subsequent Stx-mediated damage has not previously been explored.

Consequently, we aimed to examine whether the blocking of A/E lesion formation by Aurodox ultimately prevented the transversal of Stx across the epithelium, resulting in protection from Stx-mediated HUS. To explore this question, we opted to use *Citrobacter rodentium* Stx2_dact_ (Cr Stx2_dact_), an engineered *C. rodentium* transduced with a Stx2_d-_encoding prophage. For these studies, BALB/c mice were used as they do not typically reach terminal infection until 10–12 dpi, mimicking the typical timeline of HUS onset in humans and providing a window of opportunity for Aurodox treatment. Furthermore, female mice were used throughout this study as consistent weight loss and HUS-associated pathologies were not achieved using male mice (Supplementary Figs. S1 and S2).

**Fig. 2:**
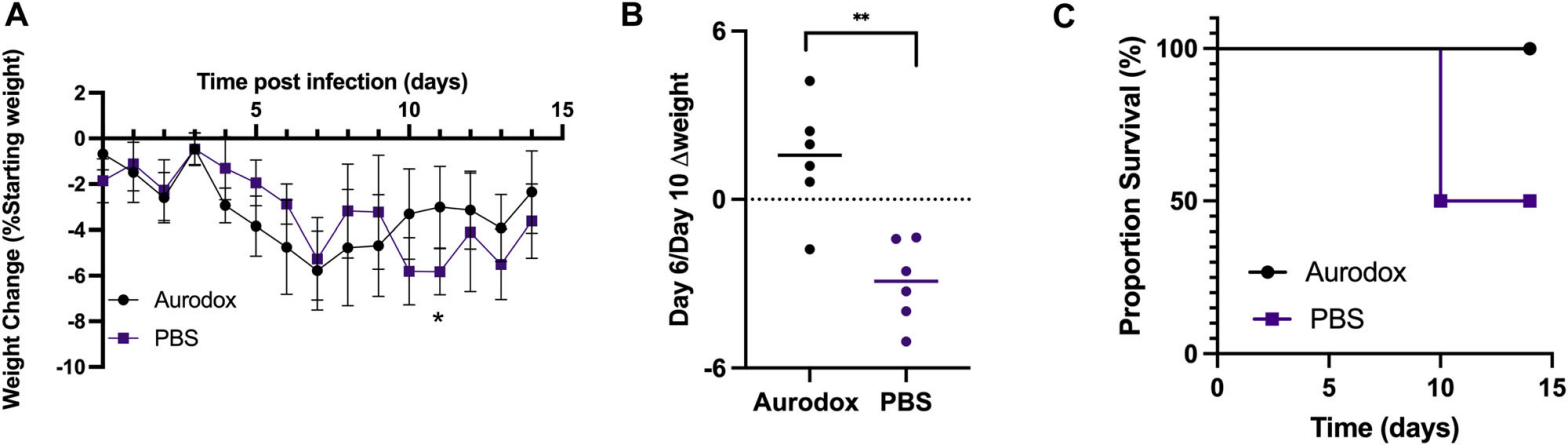
Summary of the effects of Aurodox in a murine model of STEC-HUS. **A** Percentage weight change between of Aurodox-treated vs PBS-treated animals. **B** Difference in percentage weight change of animals on Day 6 (treatment completion) and Day 10 of Aurodox vs Phosphate-Buffered Saline (PBS)-treated mice. * indicated *p* < 0.05 as determined by paired t-test. **C** Survival plot of all treatment groups.

To test this hypothesis, twelve mice were infected with Cr Stx2_dact_ (10^9^ CFU /dose). Six mice, housed in two cages of three animals, to account for cage effects, were treated daily with 25 mg/kg Aurodox by oral gavage (in 100 µl of corn oil). Six equivalent control mice were dosed xdaily with 100 µl PBS. During the dosing period (0–6 dpi), animals in both Aurodox-treated and vehicle-treated groups experienced consistent weight loss (Fig. 2A). However, the majority of Aurodox-treated mice (5/6) regained weight by Day 10. Contrastingly, all six of the PBS-treated animals continued to lose weight until Day 10 (Fig. 2B). On Day 11, three mice from the PBS-treated mice experienced worsening weight loss and clinical symptoms and were euthanised according to the conditions of our Home Office licence (Fig. 2C). These results demonstrate that Aurodox treatment protects mice against Cr Stx2_dact_- mediated weight loss, and improves survival by 50%.

### Aurodox reduces faecal shedding and histological markers of colonic hyperplasia and haemolytic syndrome in mice infected with Cr Stx2_dact_

**Fig. 3:**
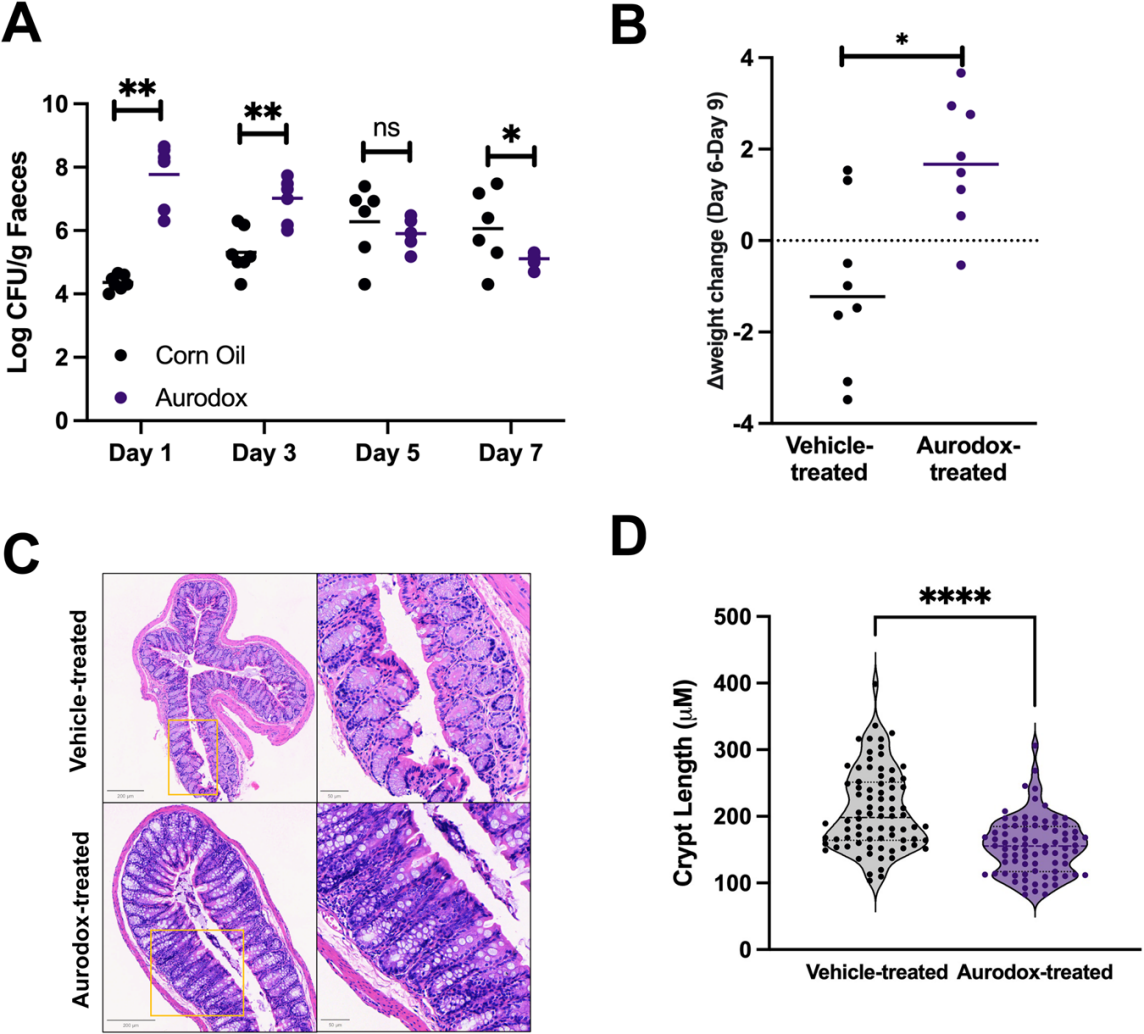
Effect of Aurodox on faecal shedding and histological markers of C. rodentium in the distal colon. **A** Faecal shedding in Aurodox-treated (n = 8) versus PBS-treated mice (n = 8) over seven days. **B** Change in weight of Aurodox-treated vs corn oil-treated mice between day 6 and day 10. **C** H & E stained sections of colons recovered from Aurodox versus PBS-treated mice, orange box shows area displayed at higher magnification. **D** Violin plot depicting crypt length of Aurodox-treated mice vs vehicle-treated mice at 10 dpi. Ten measurements were taken from two colon sections per animal (n = 80 per group). * indicates p < 0.05, **indicated p < 0.005 and *** indicated p < 0.001 as determined by paired t-test.

To gain further insight into the mechanism behind the protective effects of Aurodox, an additional Cr Stx2_dact_ oral challenge experiment was conducted. Here, eight mice, housed in groups of four per cage, were treated with Aurodox, with equivalent animal numbers allocated to control groups. In this experiment, mice in the mock-treated group were dosed with corn oil to account for the potential effects of additional dietary fat on the infection. The impact of Aurodox on colonisation was determined by measuring faecal shedding of Cr Stx2_dact_ on alternative days. This revealed that at 1 days post infection (dpi), Aurodox-treated mice shed significantly more Cr Stx2_dact_ than those treated with the corn oil vehicle. Encouragingly, faecal shedding in the Aurodox-treated mice gradually reduced over time. On the contrary, colonisation in the control mice increased over time (Fig. 3A). By five dpi, faecal shedding in the vehicle-treated mice (mean log CFU/g faeces) had exceeded that of the Aurodox-treated group, for which colonisation continued to decline. It was therefore hypothesised that through blocking the T3SS, Aurodox may encourage shedding the initial inoculum in the preliminary period following infection. Furthermore, Aurodox-treated mice regained weight between the suspension of dosing (Day 6) and peak infection (Day 9), a consistent observation in all experiments.

To corroborate these results, the ability of Aurodox to block pathological features of the colon disease associated with *C. rodentium* infection was examined. Distal colons, previously shown to be targeted by *C. rodentium* for colonisation, were harvested for histopathological analyses (H & E staining). Analysis of scanned histology slides and 40x magnification revealed extensive hyperplasia in colons from vehicle-treated mice. Contrastingly, hyperplastic regions in colons from Aurodox-treated mice were sparse (Fig. 3C). To quantify this, crypt length was measured, revealing that the median crypt length of vehicle-treated mice was significantly larger than to those treated with Aurodox (p = 0.0039, Fig. 3D). In vehicle-treated mice, characteristic goblet cell depletion was observed. This a known phenomenon associated with the murine B/T cell response to *C. rodentium* infection, and is associated with tissue recovery^30^. These results confirm the protective effects of Aurodox against the enteric pathologies of *C. rodentium*.

### Aurodox protects mice from renal pathologies associated with Stx-mediated HUS

**Fig. 4:**
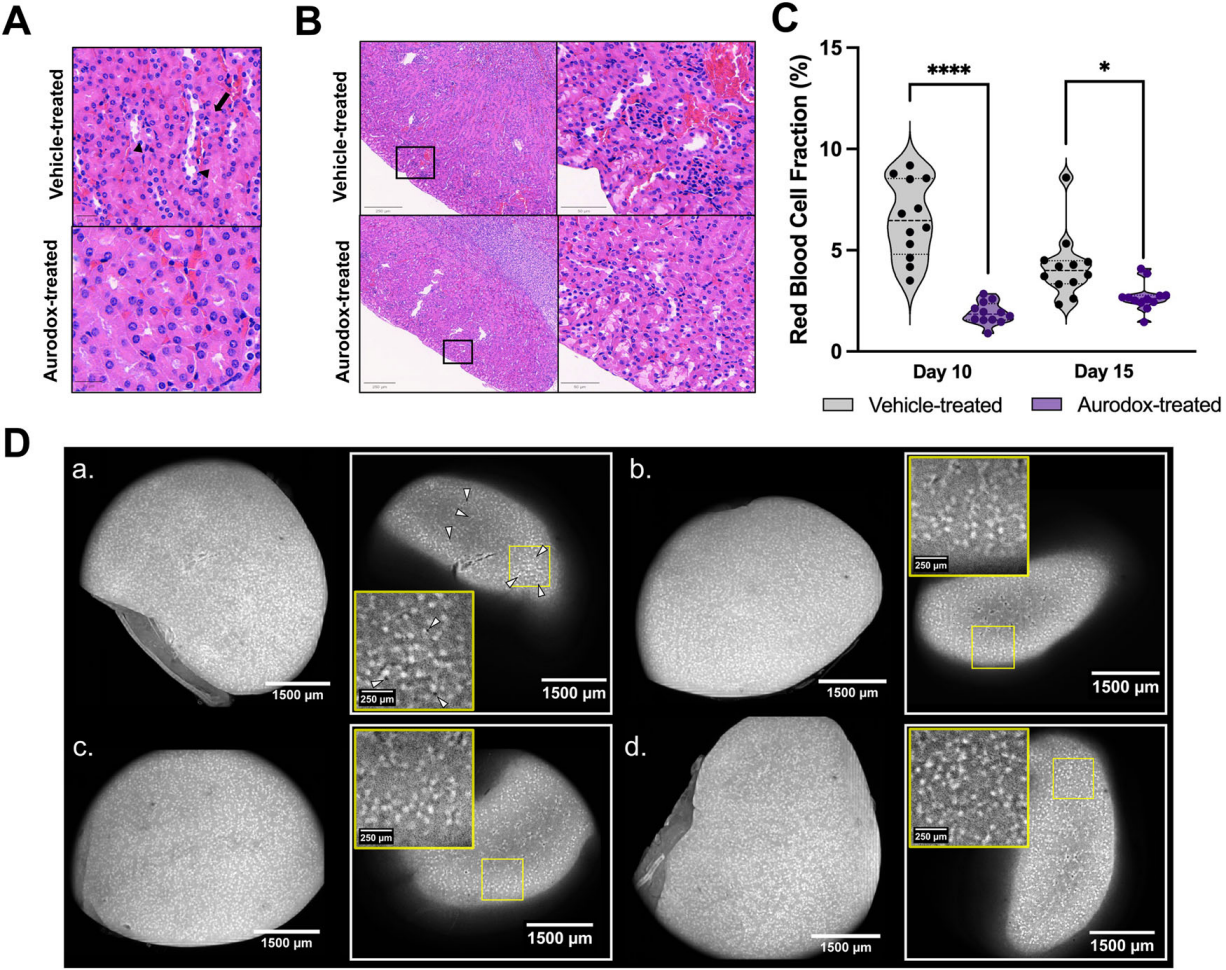
Effect of Aurodox on kidneys of Cr Stx2_dact_ -infected mice. **A**, **B** Images of H&E-stained slides from Aurodox vs PBS-treated mice sacrificed on Day 10 (peak infection). **C** Violin plot displaying red blood cell fraction of kidneys from Aurodox-treated mice vs vehicle-treated. **D** Optical clearing and Mesolens imaging of whole kidneys from (a) vehicle-treated mice harvested on Day 10, (b) Aurodox-treated mice from Day 10 (c) vehicle-treated mice harvested on Day 14 (d) Aurodox-treated mice on Day 14. Yellow boxes indicate magnified area.

Distinct indicators of Stx-mediated renal damage can be identified by histopathological analysis of kidney tissue biopsies from HUS patients. These include sloughing of necrotic luminal cells, loss of lining epithelium, tubular dilation and interstitial haemorrhaging of blood vessels. Mallick et al. described the recapitulation of these pathologies in kidneys harvested from mice infected with Cr Stx2_dact._ To gauge whether Aurodox could prevent the development of this acute renal damage in mice, kidney sections harvested from mice 10 (peak of infection) and 14 dpi respectively (recovering mice) from the experiment described in Fig. 3 were also subject to histopathological analysis. Extensive renal damage was identified in the vehicle-treated animals, including widespread loss of tubular epithelium and sloughing of epithelial cells into the lumen (Fig. 4A). Proximal and distal tubules remained largely intact for Aurodox-treated mice. Extensive interstitial haemorrhaging was also observed in vehicle-treated mice culled at 10 dpi (Fig. 4B). To quantify this, a custom object analysis pipeline was applied (Supplementary Fig. S1). Overall red blood cell proportion was significantly higher in vehicle-treated mice than in those treated with Aurodox, indicating a reduction in interstitial haemorrhaging (Fig. 4C).

During STEC-HUS, Stx is present in the blood in both its free form and bound to blood cells including platelets and monocytes. As blood filters through the kidneys, endothelial cells of capillaries and tubules are exposed to the toxin, leading to widespread damage that is difficult to quantify using traditional histopathological techniques. We used cross-scale mesoscopic imaging to address this issue, whereby intact chemically cleared whole mouse kidneys were imaged with sub-cellular resolution throughout the capture volume. Thus, the second kidney from each mouse was subject to optical clearing and 3D confocal imaging using the Mesolens platform gauge the effects of Stx on the renal macrostructure and determine whether Aurodox could protect against these effects (Fig. 4D). In the vehicle-treated mice, significant pores were identified in the kidney at 10 dpi (Fig. 4Da). but were not present in any of the other treatment groups (Fig. 4Db, c, d). These pores were ubiquitous throughout the tissue and appear to indicate tubular dilations associated with Stx-mediated renal damage. These images confirm that Aurodox protects mice against Stx-mediated renal damage, further enhancing the potential of the compound to be used as an antivirulence therapy for STEC infections.

### Aurodox treatment modulates the murine gut microbiome

Traditional antibiotic therapy is not recommended for the treatment of STEC as it is contraindicated with increased incidence of HUS^10^. Dysregulation of the gut microbiome initiated by antibiotic treatment can also deplete the microbiota of protective strains that compete with pathogens to inhibit colonisation. It is widely proposed that antivirulence therapies would minimise these effects on the native gut microflora, due to their limited spectrum of activity. However, our current knowledge of the impact of AV drugs on the wider gut microbiota is limited. Given that Aurodox is a known bactericidal inhibitor of some Gram-positive species, it is pivotal for its clinical development that its impact on the gut microflora is evaluated. To study the influence of Aurodox on the murine gut microbiome, four experimental groups were established (*n* = 4 per group). These were mock-infected & corn oil-treated; mock infected & Aurodox-treated; Cr Stx2_dact_-infected & corn oil-treated and Cr Stx2_dact_-infected & Aurodox-treated. As before, animals were dosed with 10^9^ CFU Cr Stx2_dact_ (or PBS control) and dosed for seven days with corn oil or 25 mg/kg Aurodox. Colonisation in infected animals was also monitored, with faecal shedding trends following those of our previous experiment (Supplementary Fig. S3). Notably, no animals from any treatment group reached terminal levels of weight loss, however, as previously observed, weight in Aurodox-treated animals increased post-treatment window, whereas weight continued to decline in vehicle-treated animals.

**Fig. 5:**
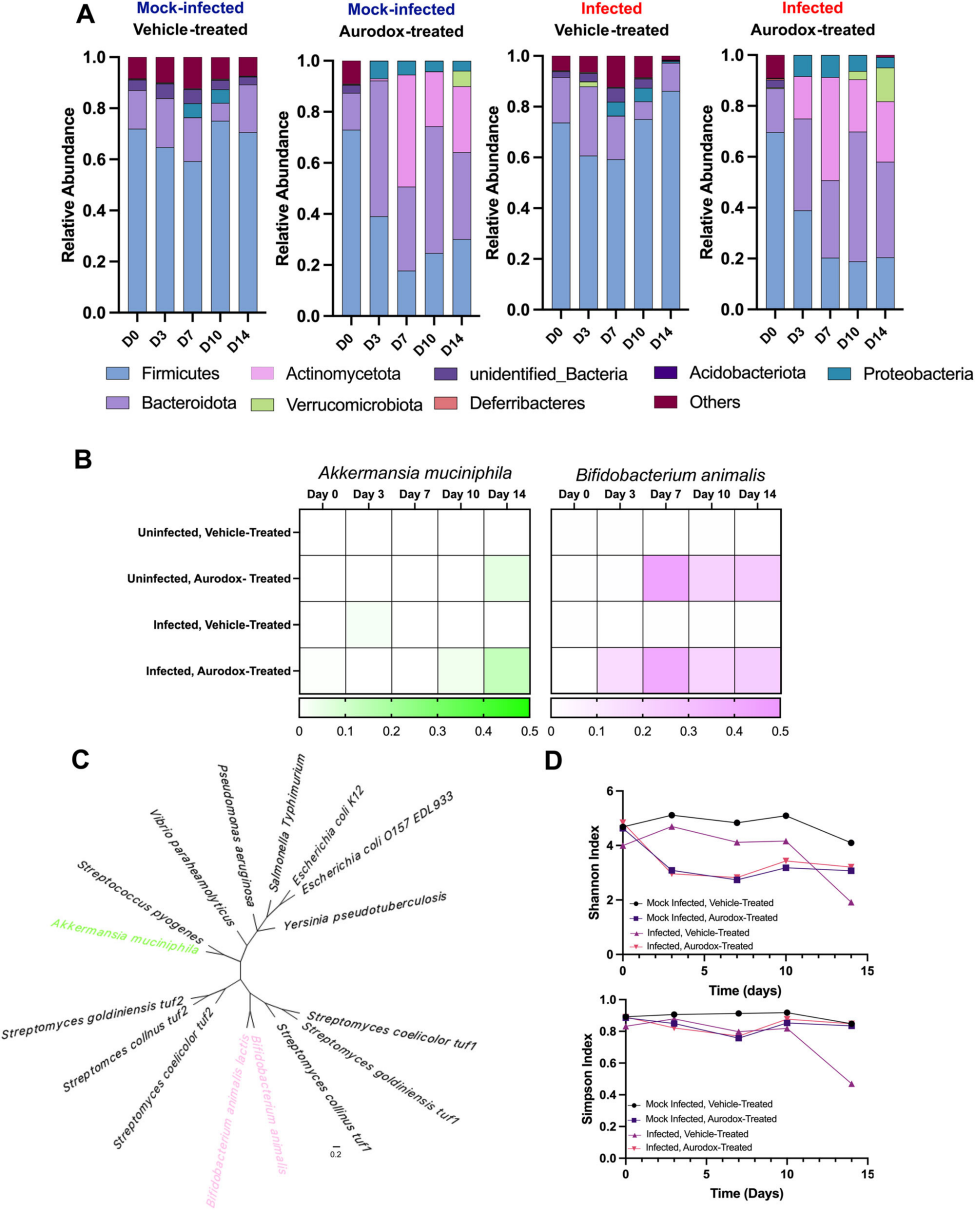
Influence of Aurodox treatment on the murine microbiota. **A** Abundance of bacterial Phyla in mock-infected, vehicle-treated; mock-infected, Aurodox-treated; Cr Stx2_dact_-infected, vehicle-treated and ; Cr Stx2_dact_-infected, Aurodox-treated mice. Relative abundance measured at five timepoints 0,3,7,1 and 14 days. **B** Heatmap depicting abundance of Akkermansia muciniphila and Bifidobacterium animals in all treatment groups, at all time points. **C** Maximum-likelihood phylogenetic tree built from Elongation Factor Thermo-unstable (EF-Tu) amino acid sequences from diverse bacterial strains. **D** Shannon and Simpson Indices of alpha-diversity in all treatment groups.

DNA was extracted from fresh faecal pellets collected on Day 0, Day 3, Day 7, Day 10 and Day 14 for and was subjected to 16S amplicon sequencing (V3-V4, Novogene). Classification of Operational Taxonomic Units (OTUs) facilitated the calculation of relative abundance at all taxonomic ranks (Supplementary Figs. S4–S9). This revealed a high degree of similarity between the faecal bacteriome of samples collected on Day 0 across all treatment groups, with 67% of species detected in all four treatment groups (Supplementary Fig. S3). At Day 0, the Firmicutes were universally the most abundant phyla (RA range 0.69 to 0.71), with high levels of *Lactobacillus* species responsible for this dominance (Fig. 5A). This consistency is reflective of the highly controlled conditions in which mice are housed.

Predictably, the microbial community within the mock-infected, mock-treated group remained stable across all time points. On the contrary, mock-infected, Aurodox-treated animals experienced a prominent shift in the microbiota. The abundance of Actinomycetota increased by >1200 fold between Day 0 and Day 7 (0.000034 to 0.438), with levels remaining elevated throughout the experiment (Fig. 4A). This increase in Actinomycetota is echoed in the Cr Stx2_dact_-infected, Aurodox-treated animals with abundance increasing from 0.0 to 0.4 in the first seven days. On the contrary, Actinomycetota abundance did not rise above 0.0001 in untreated mice regardless of infection status. Analysis of the composition of these Actinomycetota revealed that a bloom in one species, *Bifidobacterium animalis*, is responsible for this shift (Fig. 5B). As Aurodox is known to exhibit a bactericidal effect through binding Elongation Factor Thermo-unstable (EF-Tu), it was proposed that *B. animalis* may carry a resistant copy of EF-Tu, allowing it to overcome potential bactericidal effects of Aurodox, ultimately leading to community dominance. The EF-Tu gene from *B. animalis* was aligned to EF-Tu genes which are known to confer resistance or sensitivity to Aurodox. Maximum-likelihood phylogeny analysis of protein sequences revealed that EF-Tu from *B. animalis* is assigned to the same clade as Aurodox-resistant EF-Tu genes found in Aurodox-producing bacteria such as *Streptomyces goldiniensis*^31^ (Fig. 5C). Analysis of the Aurodox binding site within EF-Tu revealed changes to the consensus binding sequence consistent with Aurodox resistance (Supplementary Fig. S11). In addition, a > 6000-fold increase in Verrucomicrobiota was observed in mock-infected, Aurodox-treated groups, with a > 75-fold increase in the Cr Stx2_dact_-infected, Aurodox-treated mice. Again, one species, *Akkermansia mucciniphilia* dominated this population. These results demonstrate the capacity of Aurodox to modulate the gut microbiota in both Cr Stx2_dact_ infected and uninfected mice.

To gauge the consequences of Cr Stx2_dact_ infection and Aurodox treatment on the overall diversity of the gut microbiota, Shannon and Simpson indices of alpha diversity were calculated. According to Shannon index, vehicle-treated mice infected with Cr Stx2_dact_ experience a decline in alpha diversity following Day 10 (peak infection). Calculation of the Simpson Index corroborated this, with all groups maintaining stable diversity indices, except for the Cr Stx2_dact_-infected, untreated group (Fig. 2D). These data demonstrate that Aurodox treatment drives significant changes in the microbial population of the murine gut. However, these changes do not significantly reduce alpha diversity but conversely restore the reduction in alpha diversity initiated by the Cr Stx2_dact_ infection. Furthermore, Aurodox treatment is associated with increased abundance of species including *Bifidobacterium animalis* and *Akkermansia muciniphila* which are widely regarded as probiotic strains with many reported protective effects^32–35^.

## Discussion

Shiga Toxin-Producing *E. coli* (STEC) infections are a significant threat to public health. There are an estimated 2.8 million cases per year^36^, with modern food supply infrastructure and climate change increasing the incidence of large, multinational outbreaks^5^. Antimicrobial therapies are not currently recommended for the treatment of STEC infections, as many traditional antibiotics trigger the production of phage-derived Shiga toxins^10^. In addition, antibiotics are known to perturb the gut microbiota, which can result in dysbiosis, a major risk factor for the recurrence of infection or the emergence of opportunistic pathogens^37^. Therefore, treatment options for STEC patients are limited to supportive therapies such as total blood volume expansion (TBVE) and renal dialysis^28^. These factors highlight the urgent need for novel therapies for STEC infection.

Here, we demonstrate the efficacy of Aurodox as a potential treatment for STEC infections, showing that the compound protects mice from Cr Stx2_dact-_associated pathology, ultimately improving the survival of all treated animals. Although multiple T3SS inhibitors have been shown to inhibit WT *C. rodentium* infections in vivo, this is the first study to show that a T3SS inhibitor blocks the progression of intestinal disease to HUS. Our results build upon our previous work and on studies reported by Kimura et al.^17^ by demonstrating that Aurodox reduces both *C. rodentium*-associated hyperplasia of the gut, whilst also reducing the burden of HUS-renal damage. In future, we plan to determine whether Aurodox can be used therapeutically to treat established infections, by delaying onset of treatment until colonisation has been established (1-2 dpi). Furthermore, recent work from our group has demonstrated the capacity of Aurodox to inhibit the SPI-2 T3SS of *Salmonella enterica* subs Typhimurium, resulting in a significant decrease in macrophage persistence in vitro^21^. Thus, the efficacy of Aurodox to more broadly to protect against T3SS- mediated systemic *Salmonella* infection will be examined.

An additional focus of our work was defining the effect of Aurodox treatment on the composition of the gut microbiome. Many traditional antibiotics are associated with dysbiosis, where the reduction in diversity can increase the risk of opportunistic and relapsing infections. In recent years, studies by Mühlen et al. have explored repurposing clinically used, broad-spectrum antibiotics to treat STEC infections. The authors also utilised the Cr Stx2_dact_ murine model for studies which initially identified kanamycin, tetracycline and rifampicin as therapies associated with reduced burden of Cr Stx2_dact_ infection and diminished Stx-associated renal injury^38^. However, their most recent work has shown that treatment with these drugs caused a significant reduction in microbial diversity, reducing identified genera by >75%^39^, with the actinobacteria including probiotic *Bifidobacterium* almost eliminated after treatment. Uniquely, Aurodox does modulate the microbiota, with a bloom in *Bifidobacterium animalis* driving changes to their relative abundance, increasing to 0.34 and 0.37 on Day 14 in Aurodox-treated mice, regardless of infection status. Both *Streptomyces* and *Bifidobacterium* are members of the phylum Actinomycetota and carry related copies of EF-Tu (Fig. 4), a characterised Aurodox target. Within the Aurodox-binding site, *B. animalis* carries a serine-to-alanine mutation found in the EF-Tu genes of elfamycin producers *S. goldiniensis* and *S. collinus*^31,40^. As *Bifidobacterium* species are typically highly sensitive to antibiotic treatment^41^, this mutation in EF-Tu has likely facilitated the persistence, selection and dominance of *B. animalis* within the gut.

Diverse gut microbiomes are associated with positive outcomes for health and therefore the predominance of a single strain within a microbial community can have negative consequences^37^. Although *B. animalis* is dominant within the Aurodox-treated gut, it is present within a community of >45 additional species including multiple reported probiotic strains including *Akkermansia mucciniphila* and *Bacteriodes intestinalis*. Furthermore, *Bifidobacterium animalis* subspecies lactis is a leading commercial probiotic^42^ and multiple benefits of its presence have been identified including improvements in barrier function, pathogen resistance and epithelial integrity^42^. In mice, multiple *Bifidobacterium* species are known to inhibit enteropathogen colonisation in vivo through the production of acetate which stimulates the immune response of the gut epithelium. However, isolation of the strain for whole genome sequence is required to determine whether the *B. animalis* is capable of this acetate-mediated protection. There is also in vitro evidence that *B. animalis* inhibits both growth and toxin production in the opportunistic pathogen, *C. difficile*. Notably, the *Clostridia* were completely depleted in Aurodox-treated mice and did not recur. Therefore, despite significant changes in the microbial community following Aurodox treatment, the high abundance of *B. animalis* in the gut following Aurodox treatment potentially has beneficial outcomes for the patient. Future work should involve extending experiments beyond 14 days to characterise the capability of the microbiome to recover following Aurodox treatment, whilst also monitoring for incidence of rebound infection.

Our studies demonstrate that the Type III Secretion System Inhibitor, Aurodox, protects mice against Stx-mediated disease in a murine model of STEC. This work is the first to demonstrate the capacity of a T3SS inhibitor to alleviate Stx-mediated disease through the blockage of epithelial cell colonisation. We have also shown that the effect of Aurodox on the faecal bacteriome is distinct from traditional antibiotics; with Aurodox treatment associated with an increase in abundance of *Bifidobacterium animalis -* a known probiotic strain. These works further exemplify the therapeutic potential of Aurodox and highlight the drug as an excellent candidate for the treatment of STEC infections.

## Methods

### Animal study

All procedures were performed in strict accordance with the Animals (Scientific Procedures) Act 1986 with specific approval granted by the Home Office, UK (PPL PI440270). The University of Glasgow Animal Welfare Ethical Review Body (AWERB) also considered and approved these applications. Food and water were provided *ad libitum* and animals were kept at a constant room temperature of 20–22 °C with a 12 h light/dark cycle.

### Oral challenge of Balb/C mice with *C. rodentium* DBS770

To prepare the inoculum for the oral challenge, an overnight culture of *Citrobacter rodentium* DBS770 (Stx2_dact_) grown in 5 ml of LB broth was pelleted via centrifugation and resuspended in PBS to a concentration of 5 ×10^9^ CFU/ml. Specific Pathogen Free, Balb/C mice were either orally challenged with 1 ×10^9^ CFU *C. rodentium* DBS770 in a 200 µl volume, or an equivalent amount of PBS (uninfected). For the administration of Aurodox, 0.5 mg of Aurodox powder (BOC Sciences) was dissolved in 100 µl of corn oil (Waitrose) and administered to the animals daily for seven days. For the control animals in the experiment detailed in Fig. 2, control animals were dosed daily with the equivalent volume (100 µl) of phosphate-buffered saline (PBS). For the experiments described in Figs. 3 and 4, control animals were dosed with 100 µl of corn oil to control for the effects of additional fat on the gut microbiota.

### Monitoring of mice

Mice in all experiments were monitored daily. The weight of animals as a percentage of their starting weight was calculated. Animals which reached 90% of their body weight were provided with a soft diet to improve palatability. The overall health of animals was scored daily on a six-point scale which reflected social interaction, physical activity, piloerection, posture, lethargy and presence of loose faeces for each animal. Each characteristic was allotted a score of 0, normal; 1, minor changes; 2, medium changes; 3, major changes. Mice which approached 80% of their starting weight, or which showed consistent weight loss and scored of two or more from health monitoring were euthanised via increasing concentration of CO_2._

### Faecal shedding analysis

To estimate *C. rodentium* DBS770 levels by faecal shedding, mice were briefly removed from the cage individually, allowed to defecate, and faecal pellets were collected. As on occasion mice could not defecate, this was carried out on at least six mice per time point. Faeces were weighed and PBS was added to a final faecal concentration of 100 µg/ml. The suspension was stored at 4 °C for one hour to allow for the stool to soften before vortexing for one minute. Serial dilution of the faecal suspension was carried out, and 20 µl of each dilution was spotted on selective agar (chloramphenicol) and incubated at 37 °C overnight. Colonies were counted on the most appropriate dilution and CFU/g of faeces was calculated. Data for faecal shedding and weight loss was plotted in GraphPad Prism v9.

### Preparation of murine organs for histopathological analysis

Distal colons and kidneys were harvested from mice immediately following euthanasia. Organs were washed three times in PBS before being fixed in 5 ml of 10% buffered formalin (Sigma) overnight (4 °C). Samples were then stored in 70% EtOH and transferred to the University of Glasgow Veterinary Pathology laboratory, where samples were processed in EtOH and xylene before embedding in paraffin wax. Sections of 2 µm were cut and incubated at 60 °C before histological staining.

### Haematoxylin and Eosin staining

Sections were hydrated through graded alcohols and incubated in Gill’s haematoxylin for five minutes. Slides were then washed in H_2_O and differentiated in 1% acid alcohol, followed by further rinsing in H_2_O. The sections were incubated with Putts Eosin for five minutes, followed by rinsing in H_2_O. Sections were then dehydrated through graded alcohols into Citroclear clearing agent and coverslips were automatically placed using the Epredia ClearVue coverslipper. Once dry the sections were imaged at x40 and scanned using the MOTICEasyScan.

### Histopathological analysis of colon and kidney samples

Scanned slides were analysed using Qupath (v0.5.1) visualisation software. Representative images were taken using the snapshot function. For crypt length analysis, images of two sections from colons harvested from Day 10 were exported to ImageJ^43^ and 10 measurements were taken of each section using the measurement tool. Data was visualised and an unpaired T-test was carried out using Graphpad Prism v8.

### Kidney processing and tissue clearing for whole-organ imaging

Whole mouse kidneys were washed in sterile PBS immediately following dissection and then fixed by submersion in 4% (w/v) paraformaldehyde at 4 °C overnight. The fixative was removed and specimens were washed in 1x PBS three times, with each wash rotating at room temperature (RT) for 30 min. Fixed kidneys were then bleached in 35% (v/v) hydrogen peroxide by rotating overnight at RT. Specimens were then washed three times with 1x PBS, rotating at RT for 30 min, before treatment with 100 µM RNAse (EN0531; ThermoFisher Scientific, USA) in 1x PBS for 3 h rotating at RT. Without washing, the kidneys were transferred to labelled 5 ml tubes wrapped in aluminium foil and stained with 100 µM propidium iodide (P4864; ThermoFisher Scientific, USA) by rotating for 24 h at RT. The kidney specimens were then washed three times with 1x PBS, with each wash rotating for 30 min at RT. All subsequent steps were conducted in darkened conditions and in glass vials to prevent plastic degradation from the clearing solvents.

The fixed and stained kidneys were dehydrated stepwise through an increasing gradient of methanol (5.89596; Merck, USA) in distilled water (50%, 70%, 100%, 100%) rotating for 90 min at each stage at RT. Specimens were then treated with a 1:1 solution of 100% methanol and BABB (1:2 (v/v) benzyl alcohol (402834; Merck, USA) to benzyl benzoate (B6630; Merck, USA)), rotating overnight at RT. Finally, the tissue was placed in 100% BABB and gently rotated at RT until cleared (nominally, 48 h).

### Specimen preparation for whole-organ imaging

Cleared and fluorescently labelled whole mouse kidneys were prepared for imaging by mounting them in a custom Mesolens specimen chamber, as described by Clapperton et al.^44^ The kidneys were submerged in 100% BABB to minimise spherical aberration from refractive index mismatch. A large custom coverglass (0107999098; Marienfeld, Germany) was placed over the well of the imaging chamber before imaging with the Mesolens configured in confocal laser scanning mode.

### Confocal laser scanning mesoscopy

The Mesolens was configured for confocal laser scanning mode and oil immersion as previously described^45^. The mounted kidneys were imaged under oil immersion using Type LDF oil (Cargille, USA). Fluorescence excitation was provided via a 561 nm helium-neon laser set at 1 mW output power from a multi-line LightHub-4 laser combiner (Omicron Laserage, Germany). Propidium iodide emission signal was detected using a photomultiplier tube (P30-01; Senstech, UK), with a 550 nm dichroic filter (Thorlabs, USA) spectrally separating the incident excitation light from the fluorescence emission. A 600 nm long-wave pass filter (FEL0600; Thorlabs, USA) was placed in the emission path before the detector. The z-step size was set to 5 µm and the pixel size was set to 500 nm. Z-stack images were saved as.TIFF files for downstream processing and analysis.

### Image processing and analysis

All image processing was conducted via FIJI v1.54f^46^ and Imaris v9.8.0 (Oxford Instruments, UK) using a 64-bit Windows Server 2016 Standard operating system (v.1607) with two Intel® Xeon® Silver 4114 CPU processors at 2.20 and 2.19 GHz and 1.0 TB installed RAM.

Three-dimensional Mesolens datasets were prepared for presentation in FIJI by performing a median filter operation (σ = 5 pixels) and applying a single smooth operation to remove the contribution of spurious noise from the raw data with minimal impact on the image structure. Images were contrasted for presentation using the Contrast Limited Adaptive Histogram Equalisation (CLAHE) tool, with a block size of 127, a maximum slope of 3, and 256 histogram bins. Supplementary movies and projections were created in Imaris using the highest rendering fidelity for maximum-intensity projection visualisations

### Quantification of red blood cell fraction in kidneys

To quantify the relative decrease in haemorrhaging following Aurodox treatment, brightfield (RGB) H&E images of kidney samples were analysed using a custom object analysis pipeline (Citation: https://github.com/Liam-M-Rooney/RBC-area-fraction-histology, Supplementary Fig. S1). Three representative regions of interest (ROIs) measuring approximately 300 µm by 300 µm were selected from random areas of three separate tissue sections for each experimental condition. Each ROI was pre-processed by conducting a colour deconvolution operation in FIJI, using the default ‘H&E^2^’ parameters and retaining deconvolved red signal (containing the erythrocytes from the original data). These data were processed by an initial thresholding step to segment erythrocytes from the background and tissue using automated Yen parameters. The Analyse Particles function was used, with a lower area limit set to 5 µm^2^ to remove erroneous structures smaller than that of an erythrocyte. The performance was verified by generating an object overlay compared to the original ROI. To compare the degree of haemorrhaging between the treatment groups, the sum of the object areas identified by the previous particle analysis operation was divided by the total area of the corresponding ROI, providing the area fraction of erythrocytes per unit area of tissue. Three technical replicates from three biological replicates were compared for each condition (*N* = 9).

### 16S amplicon sequencing

DNA was extracted from fresh faecal samples collected on Day 0, Day 3, Day 7, Day 10 and Day 14 of the experiment using the DNeasy® PowerSoil ® Pro Kit (Qiagen). These samples were sent to Novogene (Cambridge, UK) for 16S metagenomic amplicon sequencing. Briefly, library preparation was carried out via amplification of the V3-V4 region of the 16S rRNA gene, (forward primer: CCTAYGGGRBGCASCAG, reverse primer GGACTACNNGGGTATCTAAT). Sequencing reads were acquired using the Illumina Novaseq 6000.

### 16S microbiome bioinformatic analysis

Paired-end reads were assigned to samples based on their unique barcode and truncated by cutting off the barcode and primer sequence. Paired-end reads were merged using FLASH (V1.2.7, http:// ccb.jhu.edu/software/FLASH/). Sequence analyses were performed by Uparse software (Uparse v7.0. 1001). Sequences with ≥97% similarity were assigned to the same OTUs. Representative sequences for each OTU were screened for further annotation. For each representative sequence, the Silva Database was used based on the Mothur algorithm to annotate taxonomic information^47^. Data analysis including the calculation of alpha and beta diversity indices was carried out using QIIME (v 1.9.9)^48^.

### Phylogenetic analysis of elongation factor thermo-unstable

A multiple sequence alignment of selected Elongation Factor Thermo-unstable EF-Tu proteins was generated using MUSCLE. This alignment was used to generate a maximum likelihood tree in IQ-Tree (Galaxy Version 2.3.5+galaxy0), with 1000 ultrafast bootstrap replicates^49^. The first 120 amino acids of this alignment, encompassing the Aurodox binding domain and GTP binding domains were visualised using the ggmsa package (v. 1.6.0)^50^.

## Supplementary information

Supplementary movie

Supplementary Figures

## Data Availability

Sequencing reads for microbiome analysis have been uploaded to NCBI under accession number PRJNA1165982. Raw histology images can be found on the University of Strathclyde PURE data portal at 10.15129/876a786c-7f44-431c-9e56-41dbad34dda8.
