# Supplementary material for: Evaluating the protective effects of Aurodox in a murine model of Shiga toxin-producing Escherichia coli
Source: NPJ Antimicrob Resist. 2025 Apr 1;3:23. doi: 10.1038/s44259-025-00094-3 (PMC11962119; doi:10.1038/s44259-025-00094-3)
Supplement: Supplementary file 2 — Supplementary Figures [file 44259_2025_94_MOESM2_ESM.pdf]

Supplementary Figures

Figure S1

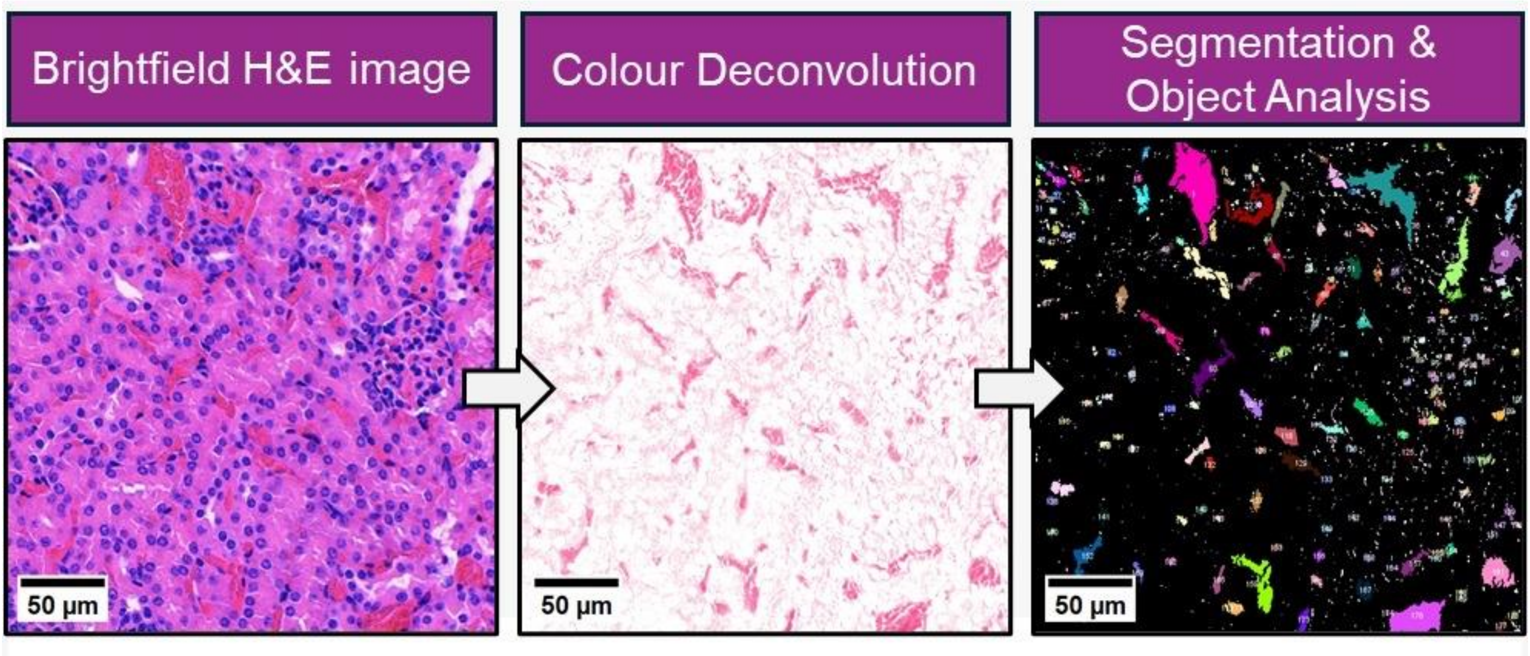

Figure S1. A pipeline for the quantification of haemorrhaging from histological brightfield microscopy data. Following acquisition of H&E-stained specimen via brightfield microscopy (RGB format) (LEFT), the erythrocytes are separated using a *Colour Deconvolution* operation in FIJI (parameters: H&E2). The erythrocytes are presented in Channel 2 of these data (CENTRE), which were then selected as discrete objects by intensity-based thresholding and segmentation. Object analysis then revealed the morphological descriptors for erythrocytes in each image prior to comparing to the tissue area to provide a comparable measurement of haemorrhaging between experimental conditions.

Figure S2

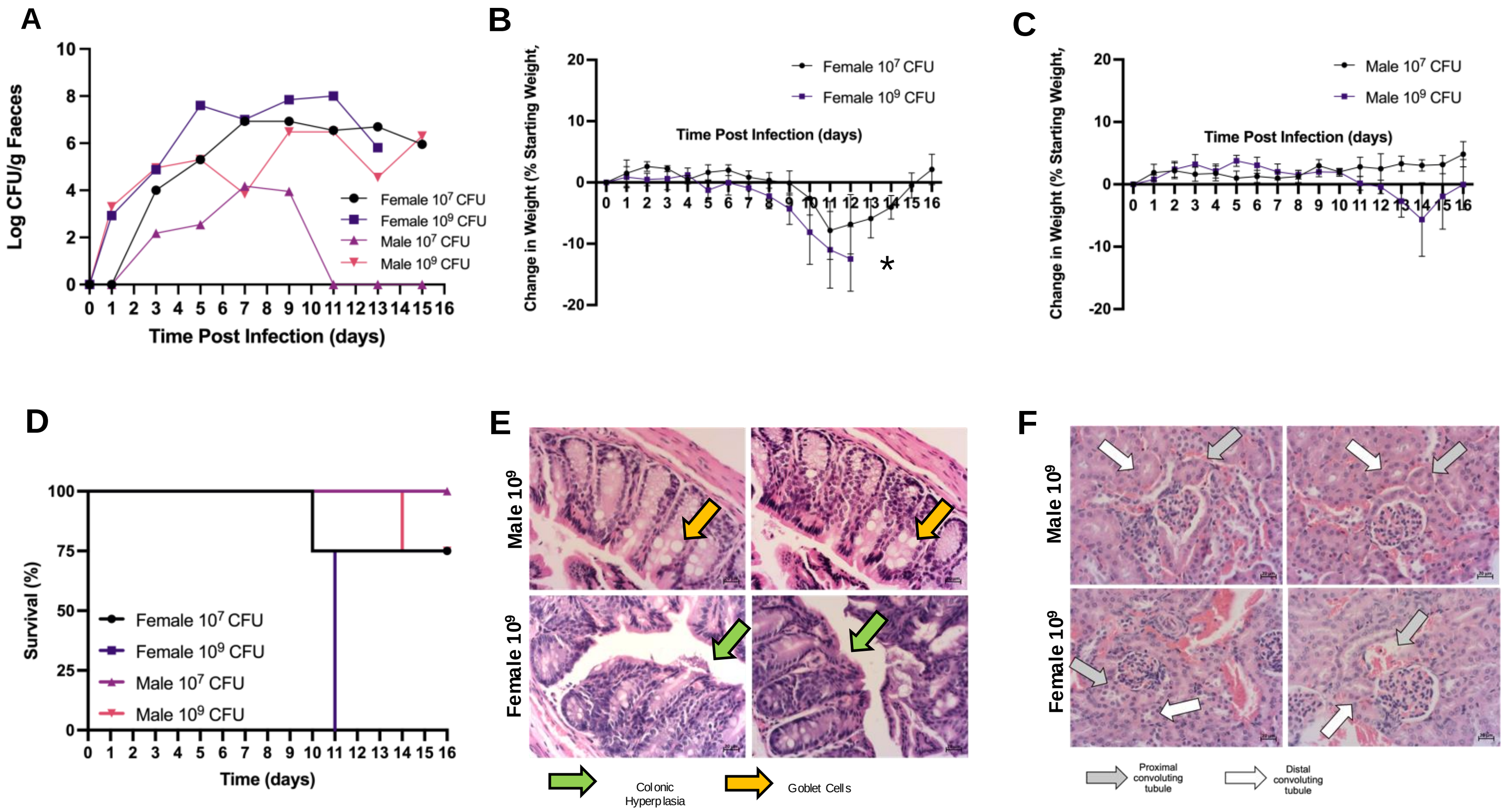

Figure S2**:* **Comparison of Male and Female Balb/C Mice in *C. rodentium* + Stx (DBS770) model of STEC-HUS.** (A) Faecal shedding of male and female mice challenged with 10^7/10^9 CFU *C. rodentium* DBS770 (B) Weight change in female mice, and (C) male mice. Experimental endpoint as determined by weight loss threshold and clinical score is indicated by **. (D) Survival plot male and female mice challenged with *C. rodentium* DBS770. (E) H&E-stained colon and (F) kidney sections from Male 10^9 group and Female 10^9 group. Green arrows indicate colonic hyperplasia, orange arrows highlight examples of goblet cells. In kidney images, arrows indicate examples of proximal convoluting tubules (grey) and distal convoluting tubules (white) with epithelial sloughing. Blue arrows highlight interstitial haemorrhaging.

Figure S3

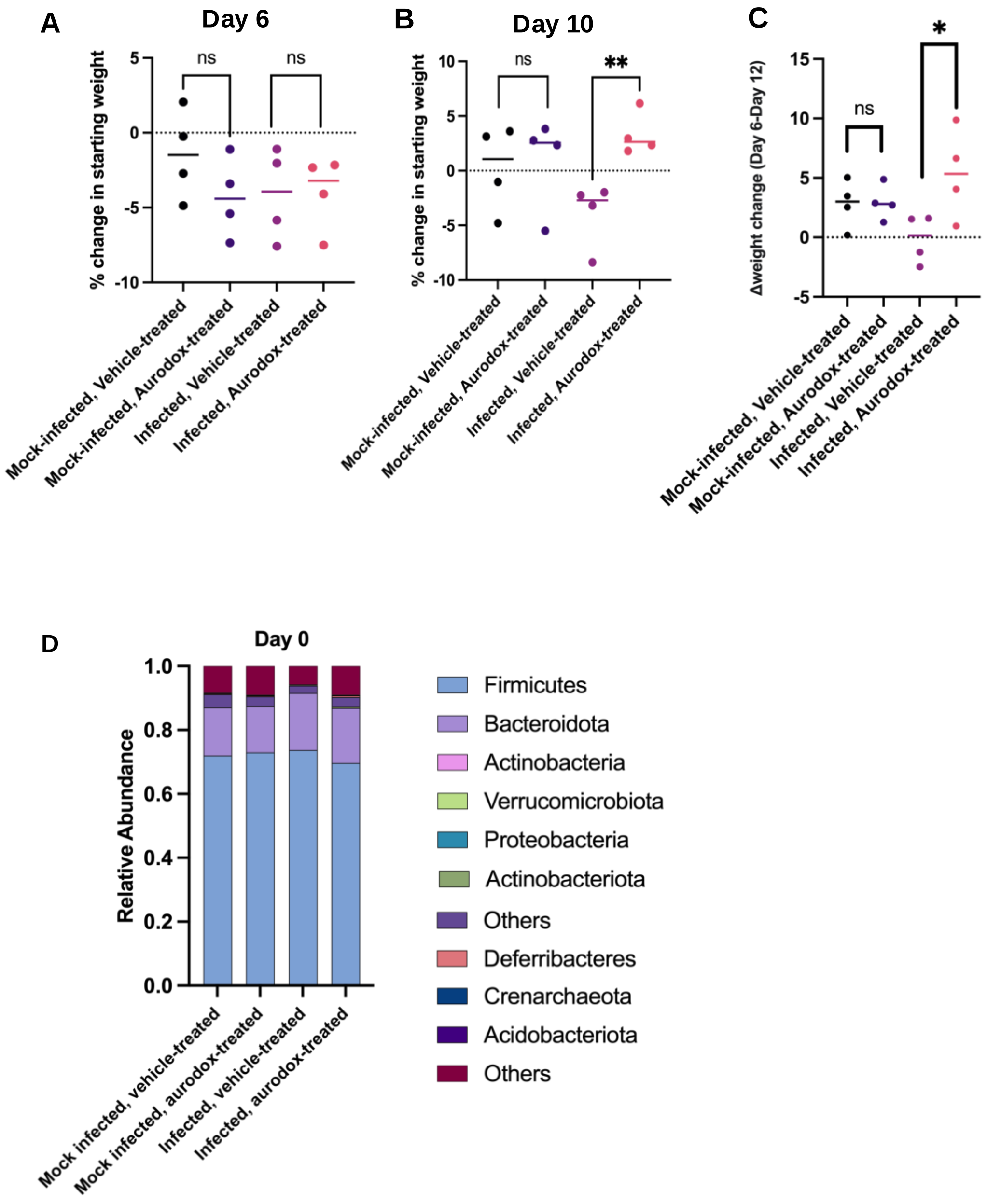

***Figure S3**: **Summary of Weight Changes in Mice Undergoing Microbiome Analysis** (A) Weight changes as a percentage of starting weight in mice mock-infected & corn oil-treated; mock infected & Aurodox-treated; Cr Stx2dact-infected & corn oil-treated and Cr Stx2dact-infected & Aurodox-treated 10^9 CFU *C. rodentium* DBS770 on Day 6 (B) Day 12 (C) Change in weight between Day 6 (end of aurodox treatment) and Day 12 (peak infection). (D) Representation of top ten most abundant phyla across four cages representing four treatment groups.

Figure S4

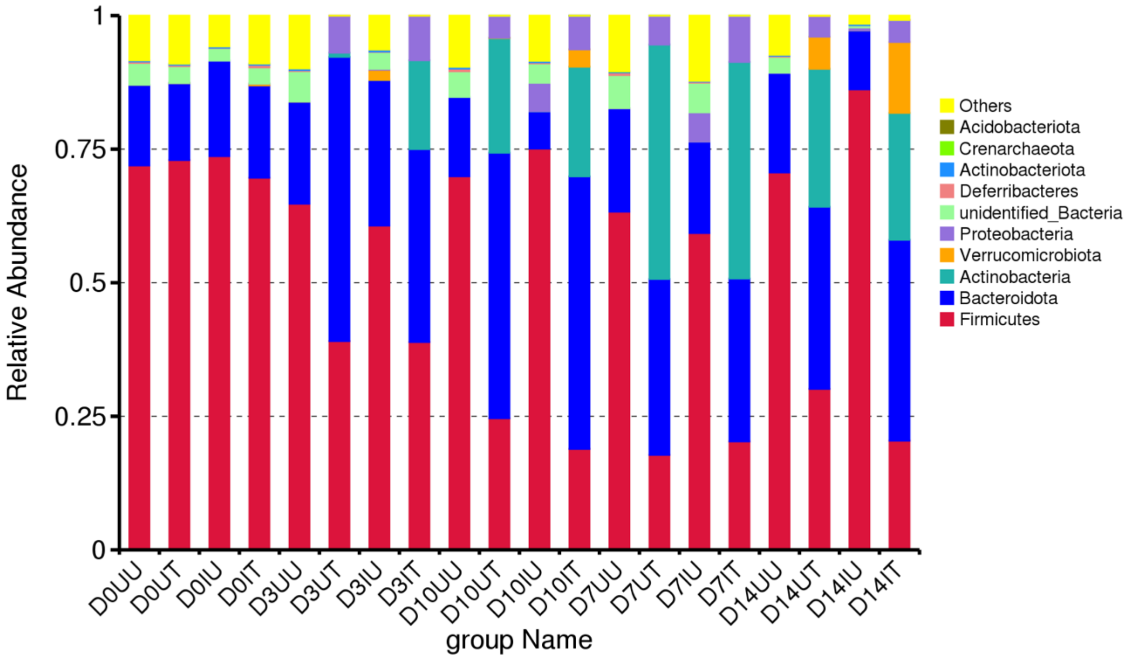

Figure S4: **Summary of Changes to Top Ten Phyla Across Treatment Groups and Time points.** Histogram representing relative abundances of top ten phyla in all treatment groups across all time points.

Phylum

Figure S5

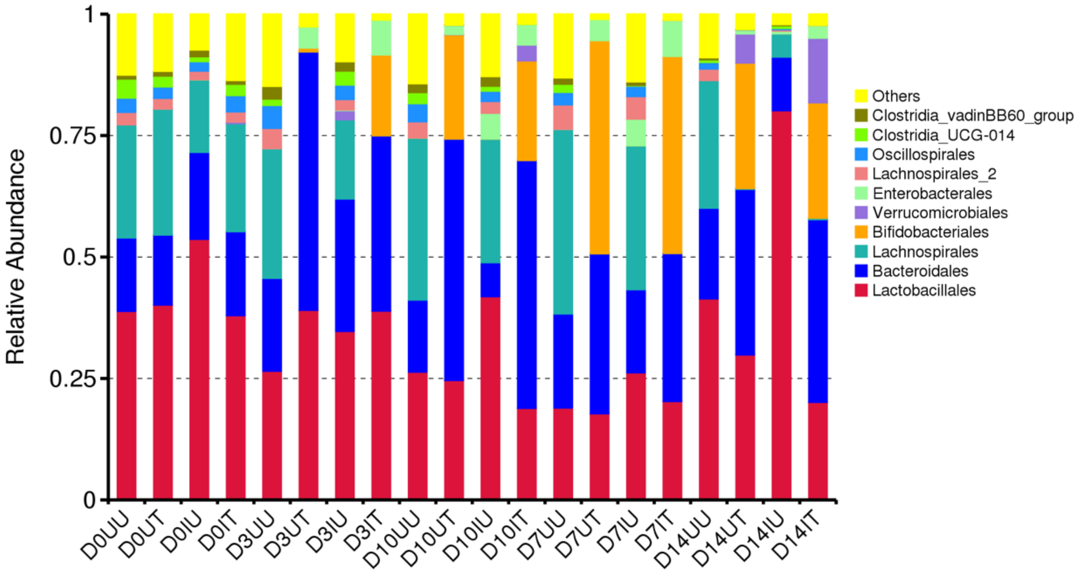

Figure S5: **Summary of Changes to Top Ten Orders Across Treatment Groups and Time points.** Histogram representing relative abundances of top ten groups in all treatment groups across all time points.

Figure S7

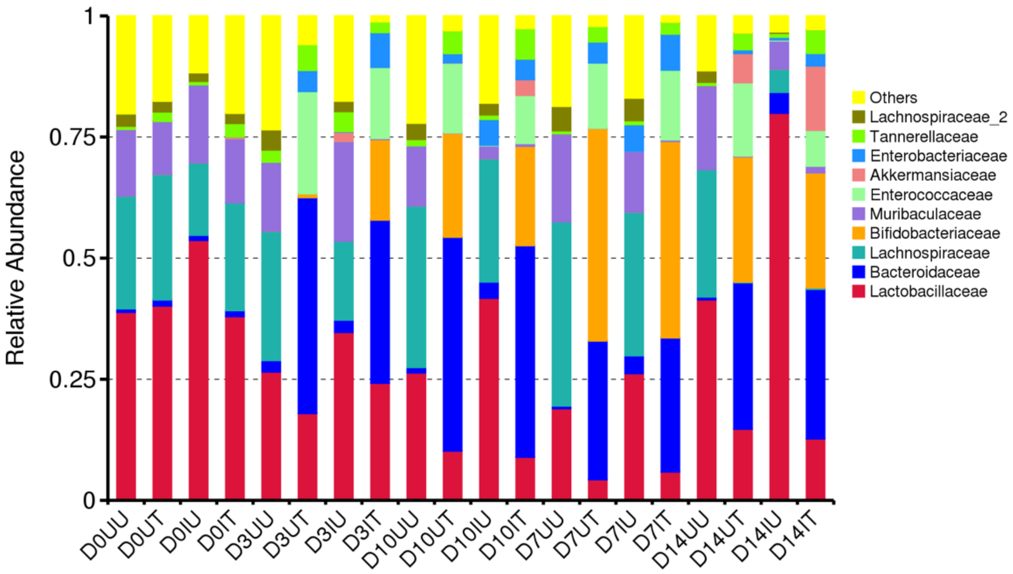

***Figure S6*: Summary of Changes to Top Ten Families Across Treatment Groups and Time points.** Histogram representing relative abundances of top ten orders in all treatment groups across all time points.

Figure S8

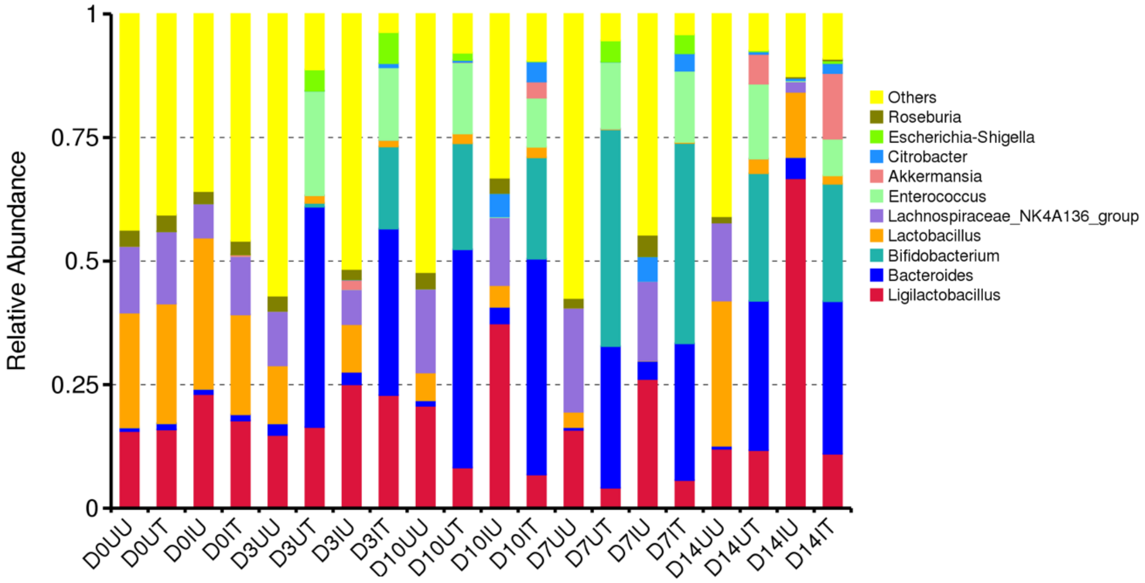

Figure S7: **Summary of Changes to Top Ten Groups Across Treatment Groups and Time points.** Histogram representing relative abundances of top ten groups in all treatment groups across all time points.

Figure S9

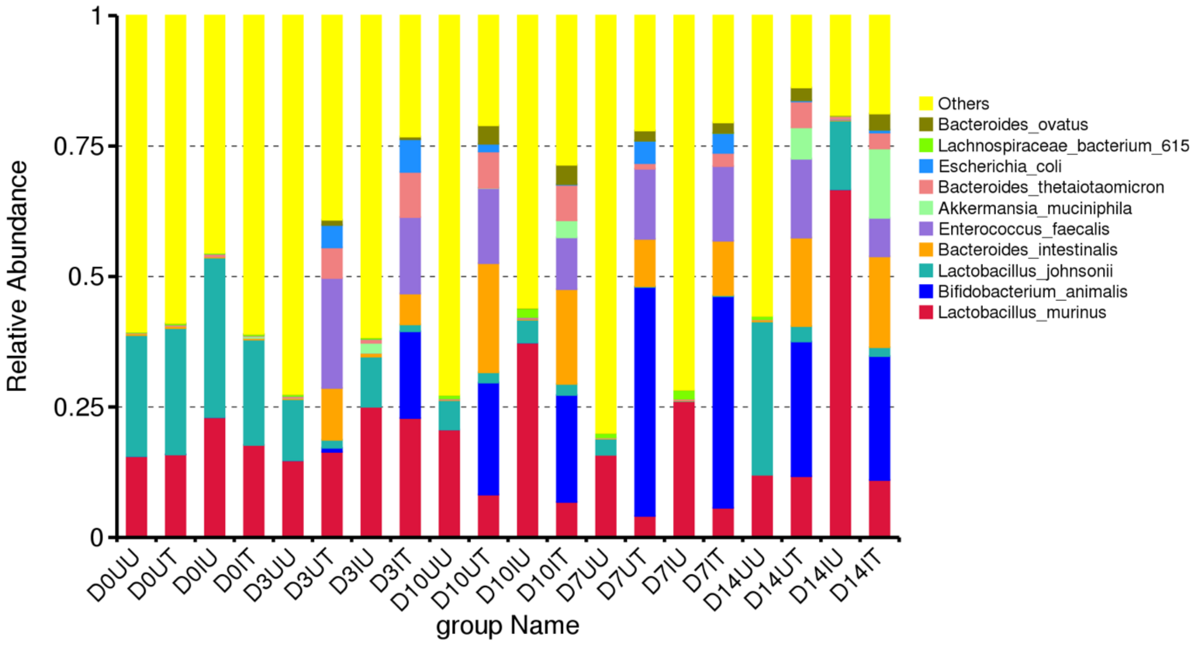

Figure S8: **Summary of Changes to Top Ten Species Across Treatment Groups and Time points.** Histogram representing relative abundances of top ten species in all treatment groups across all time points.

Table S1: Summary of Relative Abundances of Top Ten Phyla Across all Time Point and Treatment Groups.

Group	Firmicutes	Bacteroidota	Actinobacteria	Verrucomicrobiota	Proteobacteria	unidentified	Deferribacteres	Actinobacteriota	Crenarchaeota	Acidobacteriota	Others
D0UU	0.719383	0.151132	0.000009	0.000043	0.000034	0.04078	0.002856	0.002178	0	0	0.083585
D0UT	0.729674	0.144117	0.000034	0.000009	0.000137	0.031278	0.002419	0.00223	0	0	0.090102
D0IU	0.736895	0.178962	0.000009	0	0.000077	0.023148	0.000695	0.002573	0	0	0.057641
D0IT	0.696424	0.172847	0	0.003302	0.00012	0.030317	0.004768	0.002419	0	0	0.089803
D3UU	0.64783	0.191226	0.000043	0.000154	0.000077	0.056852	0.001878	0.003027	0	0	0.098913
D3UT	0.390669	0.532024	0.007804	0	0.069417	0	0	0	0	0	8.60E-05
D3IU	0.60693	0.272676	0.000026	0.018971	0.001732	0.031544	0.000849	0.003585	0	0	0.063687
D3IT	0.388997	0.361072	0.166346	0	0.083448	0	0	0	0	0	0.000137
D7UU	0.632925	0.193628	0.000034	0.000043	0.000249	0.061732	0.004923	0.001767	0.000497	0.000077	0.104125
D7UT	0.177676	0.32934	0.438714	0	0.054237	0.000009	0	0	0	0	2.40E-05
D7IU	0.592907	0.171123	0.000077	0	0.054863	0.055455	0.002101	0.001389	0	0	0.122085
D7IT	0.202967	0.304828	0.405437	0	0.086681	0.000009	0	0	0	0	7.80E-05
D10UU	0.69928	0.148431	0.000043	0	0.000137	0.047847	0.005129	0.003096	0	0	0.096037
D10UT	0.246261	0.497041	0.214528	0.000652	0.041381	0.000017	0	0	0	0	0.00012
D10IU	0.751038	0.069854	0.000154	0	0.053182	0.036304	0.002007	0.002813	0	0	0.084648
D10IT	0.188902	0.510214	0.205069	0.032633	0.062967	0.000009	0.000009	0.000017	0	0	0.00018
D14UU	0.706226	0.186672	0	0	0.000206	0.029906	0.001724	0.00187	0	0	0.073396
D14UT	0.301295	0.340815	0.25825	0.060111	0.038971	0.00006	0.000069	0	0	0	0.000429
D14IU	0.861895	0.110463	0.000051	0.000009	0.00548	0.004048	0.000094	0.002479	0	0	0.015481
D14IT	0.204271	0.375969	0.237564	0.132873	0.041012	0.00024	0.000051	0	0	0	0.00802

Figure S11

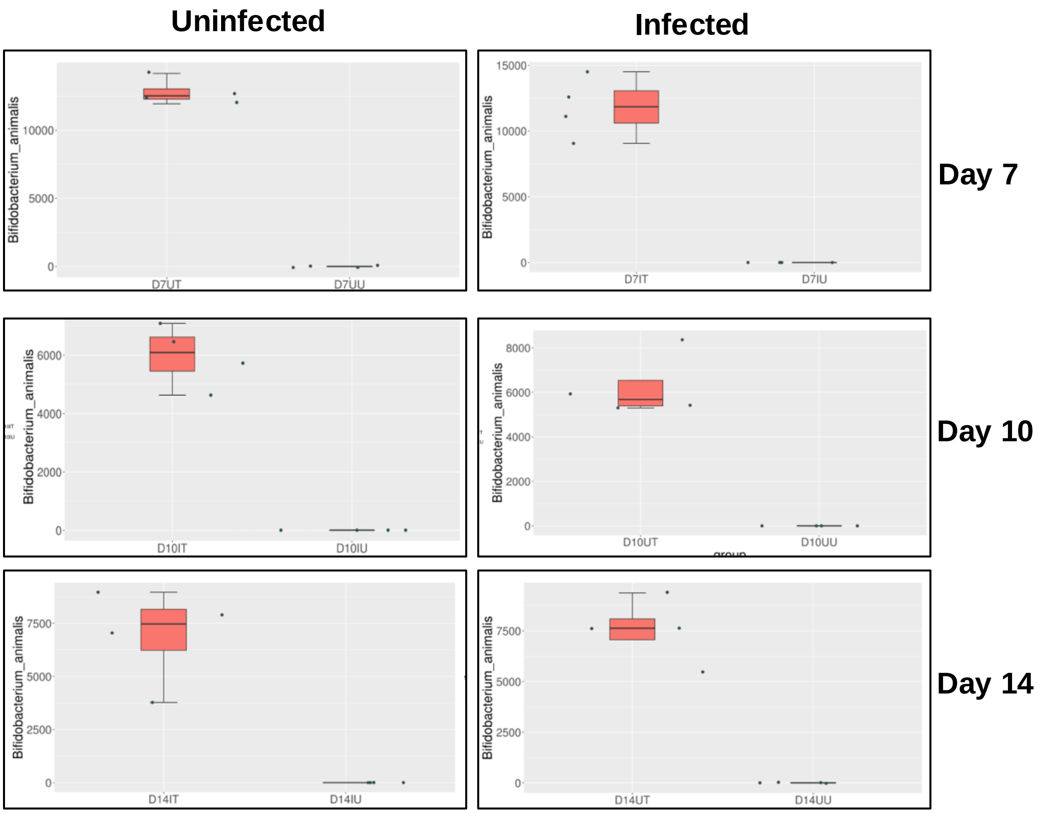

***Figure S9**:* **Summary of Variation between *Bifidobacterium animalis* Across Time Points and Treatment Groups.** Bars represent the number of OTU assignments per sample in both infected and uninfected mice at Day 7, Day 10 and Day 14.

Figure S12

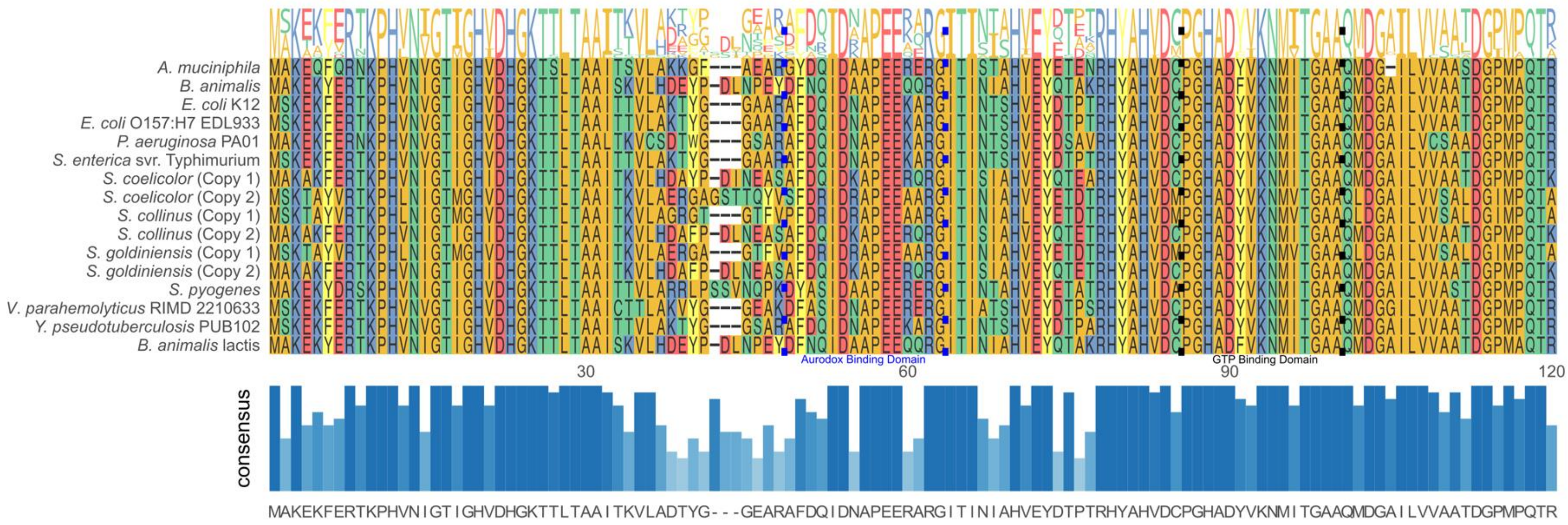

***Figure S10*: Comparison of Elongation Factor Thermo-unstable Amino Acid Sequences.** Alignment carried out using MUSCLE. The first 120 amino acids of EF-Tu sequence encompassing the Aurodox binding domain and GTP binding domains were visualised using the ggmsa package (v. 1.6.0).
